# Recurrent Non-surgical Pneumoperitoneum Associated With Jejunal Diverticulosis: A Case Report and Diagnostic Challenge

**DOI:** 10.7759/cureus.113093

**Published:** 2026-07-21

**Authors:** Katarzyna J Mazur, Amaar Aamery, Mohammad Salem Amer, Mohammed Gaafer Elamin Mohammed, Hani AL Qadhi

**Affiliations:** 1 Medicine, Jan Kochanowski University, Kielce, POL; 2 General Surgery, Sultan Qaboos Hospital, Salalah, OMN; 3 Radiology, Sultan Qaboos Hospital, Salalah, OMN

**Keywords:** conservative management, diagnostic challenge, jejunal diverticulosis, pneumoperitoneum, spontaneous pneumoperitoneum

## Abstract

Spontaneous pneumoperitoneum presents a significant diagnostic challenge because the radiological presence of free intraperitoneal gas conventionally prompts urgent clinical assessment for suspected viscus perforation. Jejunal diverticulosis is an infrequent but documented etiology of recurrent, non-surgical free air that can masquerade as a surgical emergency. A 69-year-old female patient with a three-year history of recurrent pneumoperitoneum and documented jejunal diverticulosis presented with abdominal pain and low-grade fever. Clinical examination revealed mild abdominal distension without evidence of peritonitis. Laboratory investigations identified elevated C-reactive protein alongside severe hypothyroidism, while the white blood cell count remained within normal physiological limits. Although initial computed tomography (CT) demonstrated massive free air, subsequent oral contrast CT provided additional evidence against an active perforation, supporting conservative management when interpreted alongside the patient’s stable clinical examination. Rapid clinical resolution was achieved, and the patient remained asymptomatic throughout the follow-up period. Recognizing jejunal diverticulosis as a potential cause of benign pneumoperitoneum is essential to avoid unnecessary surgical morbidity. A tailored approach, prioritizing clinical stability and nuanced imaging interpretation, is necessary to manage such cases effectively.

## Introduction

Pneumoperitoneum, defined as the presence of free gas within the peritoneal cavity, usually indicates an underlying hollow viscus perforation. While historical literature suggests that approximately 85% to 95% of such cases are associated with surgical emergencies, these figures require careful interpretation within contemporary clinical contexts and may vary across different patient populations [1,2]. Spontaneous pneumoperitoneum (SP) represents a distinct clinical entity where extraluminal gas exists without evidence of visceral perforation [1,3]. This condition frequently presents a significant diagnostic challenge for surgeons, as radiographic subdiaphragmatic air can masquerade as a surgical emergency [4-6]. Consequently, certain retrospective studies have identified negative findings in approximately 20% to 31% of exploratory laparotomies performed for suspected perforations [2,6]. To minimize the risk of unnecessary surgical morbidity, the determination of the management strategy must be dictated by the patient’s overall clinical stability and physical examination findings rather than relying exclusively on the presence of gas on imaging studies [1,2,6]. The etiology of SP is remarkably diverse and is generally categorized into intrathoracic causes (e.g., barotrauma from mechanical ventilation, cardiopulmonary resuscitation, or chronic obstructive lung diseases), intra-abdominal origins (most notably pneumatosis cystoides intestinalis), gynecological pathways (where air enters the coelomic cavity via the fallopian tubes during pelvic manipulation or specific postpartum activities), and idiopathic [7,8].

Jejunal diverticulosis is a rare clinical entity compared to the more prevalent colonic diverticula [4,9]. The reported incidence of jejuno-ileal diverticulosis is low, ranging from 0.1% to 2.3% in the general population, although some autopsy studies suggest rates up to 4.5% [4,10]. These are typically acquired "pulsion" diverticula, involving the protrusion of mucosa and submucosa through weakened areas of the muscularis at the entry points of mesenteric vessels [9]. Most cases are asymptomatic, while others may experience only vague, non-specific gastrointestinal symptoms [9,10]. Despite its usually benign nature, complications such as diverticulitis, hemorrhage, or perforation can occur, leading to significant morbidity [4,10]. 

Jejunal diverticulosis represents an uncommon but established etiology of chronic or recurrent SP [1,11]. The underlying pathophysiology remains a subject of scientific speculation, with hypotheses focusing on either transmural gas equilibration through thinned diverticular mucosa or the occurrence of subclinical microperforations that fail to induce significant enteric contamination [1,11]. This phenomenon introduces substantial complexity into surgical decision-making, particularly when imaging reveals extensive pneumoperitoneum in a hemodynamically stable patient [4]. This case report details a distinctive clinical course characterized by a multi-year history of recurrent free air episodes and a prior diagnostic laparoscopy that definitively identified extensive diverticula without associated perforation. Despite the radiographic evidence of massive pneumoperitoneum during the most recent admission, the patient exhibited limited clinical findings, which permitted a successful repeat of non-operative management. This case illustrates the diagnostic nuances of the condition while advocating for a specialized management strategy to mitigate the risks of unnecessary surgical interventions [2,4]. 

## Case presentation

A 69-year-old female patient with a medical history of pulmonary hypertension, hypothyroidism, and a surgically corrected atrial septal defect (in 2019) presented with a three-day history of right upper quadrant abdominal pain, nausea, and constipation.

The patient presented with a baseline functional status characterized by independent mobility and a Glasgow Coma Scale score of 15. Clinical status, due to the symptoms and medical and surgical history, was consistent with an American Society of Anesthesiologists (ASA) Physical Status III [5], indicating a high perioperative risk profile that necessitated a cautious approach to surgical intervention. No history of tobacco use was documented in the clinical record.

**Figure 1:**
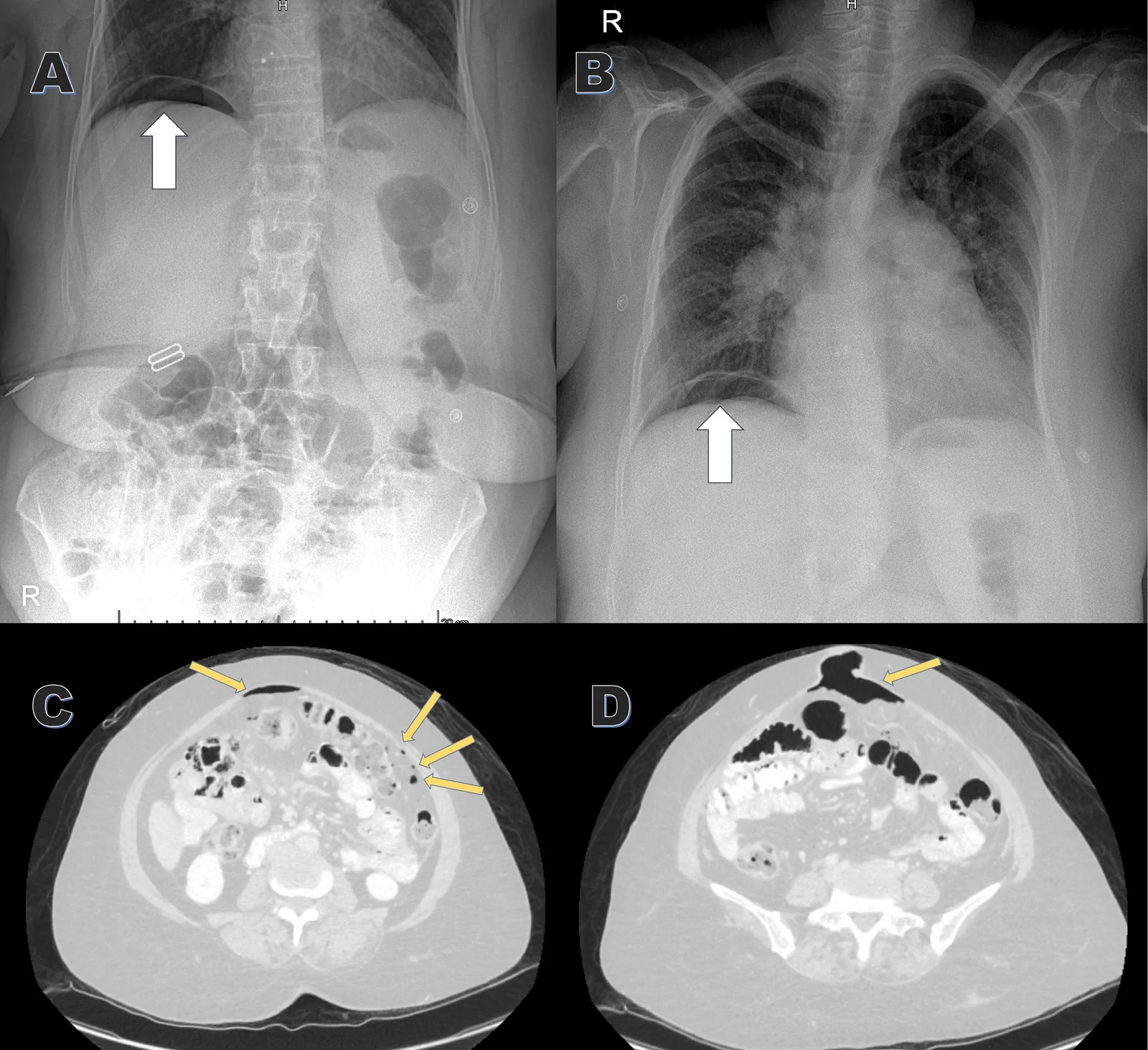
X-ray and CT images (A) X-ray of the abdomen, anteroposterior (AP) erect view; (B) X-ray of the chest, posteroanterior (PA) view, showing air under the right copula of the diaphragm (white arrows); (C-D) Contrast-enhanced computed tomography (CECT) study of the abdomen with oral contrast, axial cuts, lung window, showing evidence of pneumoperitoneum (yellow arrows). No evidence of contrast leakage or signs of acute enteric obstruction.

The patient’s surgical history included a laparoscopic cholecystectomy in 2021 and a subsequent diagnostic laparoscopy in late 2022. This surgical intervention was necessitated by an episode of acute abdominal pain and radiographic evidence of pneumoperitoneum, as shown in Figure 1.

Intraoperative findings from the 2022 procedure revealed approximately 100 ml of free serosanguinous fluid in the pelvis and a one-meter segment of multiple jejunal diverticula originating 50 cm distal to the duodenojejunal flexure. Although mild peridiverticular inflammation was observed, careful examination of the entire small bowel from the duodenojejunal flexure to the ileocecal junction confirmed the absence of visceral perforation. Additionally, the surgeons identified and repaired a right hypochondriac port-site hernia and an umbilical hernia. The final postoperative diagnosis was established as multiple jejunal diverticulosis complicated by recurrent SP alongside concurrent ventral hernias. 

**Figure 2:**
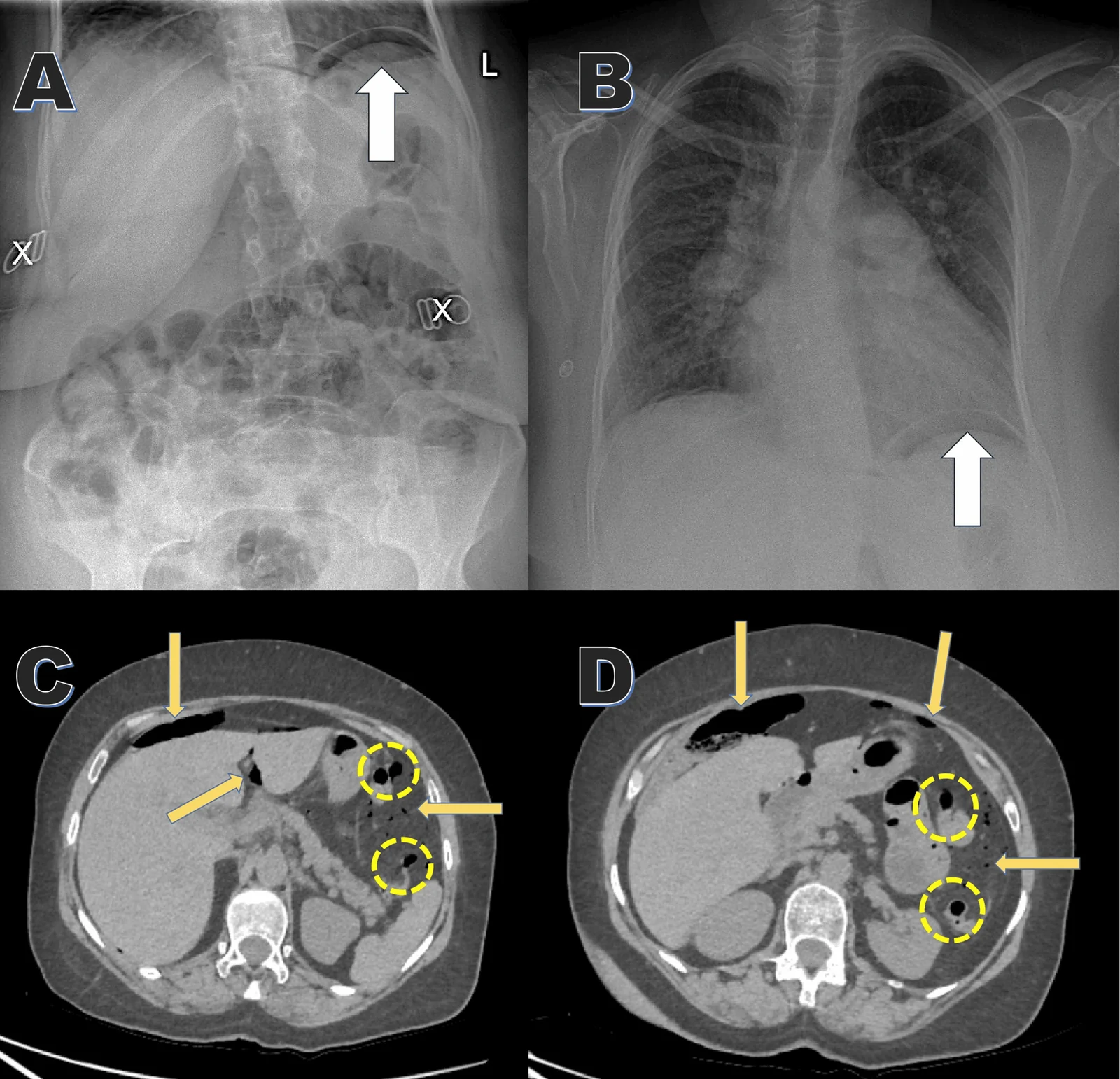
X-ray and CT images (A) X-ray of the abdomen, anteroposterior (AP) erect view; (B) X-ray of the chest, posteroanterior (PA) view, showing air under the left copula of the diaphragm (white arrows); (C-D) Plain computed tomography (CT) study of the abdomen, axial cuts, showing evidence of pneumoperitoneum (yellow arrows) and multiple jejunal diverticulae (yellow circles).

Between 2023 and early 2025, the patient required three hospitalizations for recurrent symptoms, all of which were managed through non-operative strategies until clinical stabilization was achieved. The first episode, in mid-2023, involved abdominal pain where computed tomography (CT) identified multiple jejunal diverticula without evidence of pneumoperitoneum. During the second admission, a month later, she presented with abdominal pain, and contrast-enhanced CT (CECT) revealed pneumoperitoneum with right-sided fat stranding but no contrast extravasation (Figure 2).

**Figure 3:**
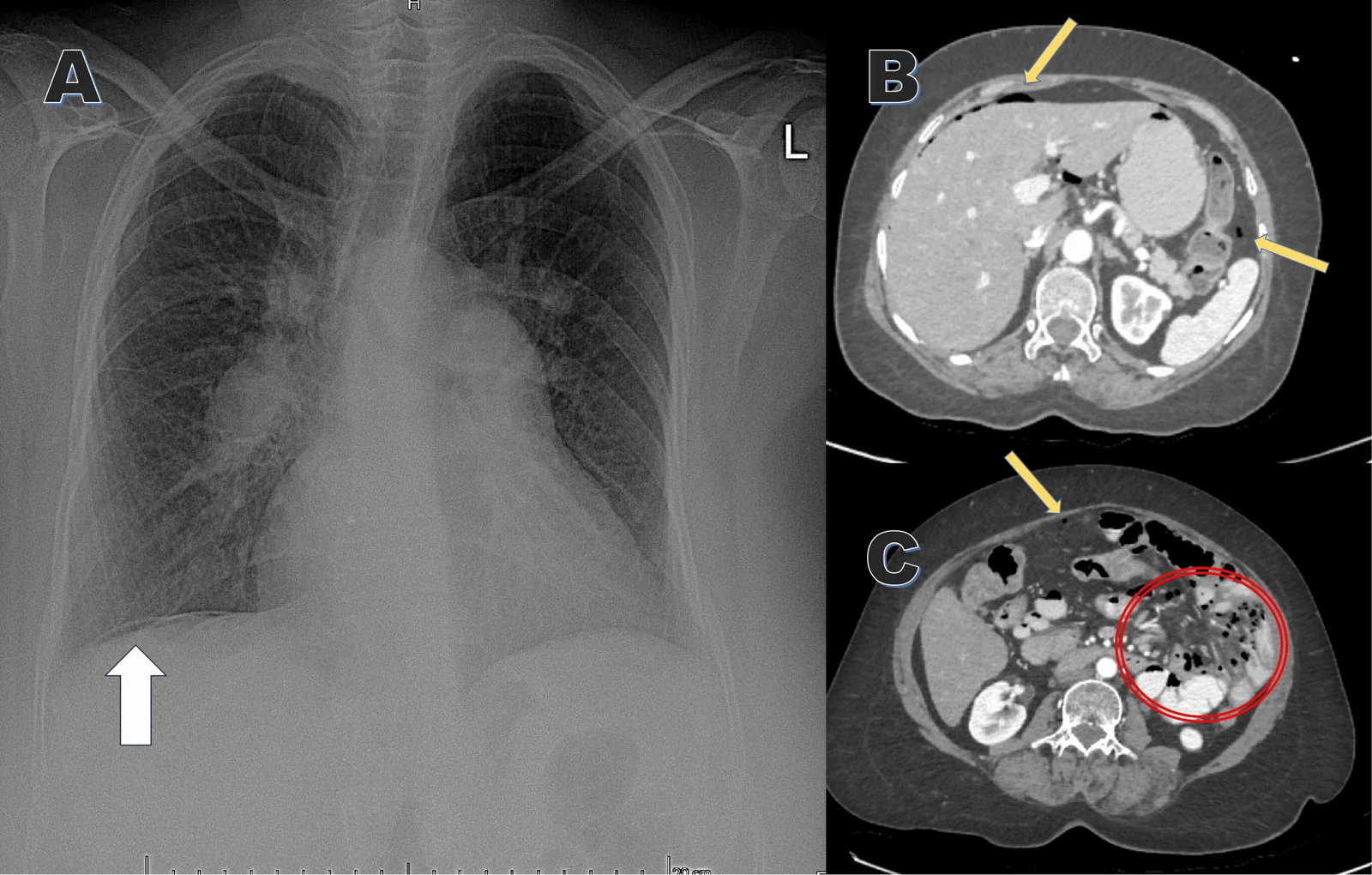
X-ray and CT images (A) X-ray study of the chest, posteroanterior (PA) view, showing subtle air under the right copula of the diaphragm (white arrow);  (B-C) Contrast-enhanced computed tomography (CECT) study of the abdomen with oral contrast, axial cuts, showing evidence of pneumoperitoneum (yellow arrows) and multiple jejunal diverticulae (red circle). No evidence of contrast leakage or signs of acute enteric obstruction.

The third recurrence occurred less than two years later, characterized by right-sided abdominal pain and imaging findings of mild pneumoperitoneum alongside gas vacuoles clustered near the jejunal loops (Figure 3).

Although she demonstrated clinical improvement following conservative treatment during each stay, the patient did not attend the recommended outpatient diagnostic workup, which was to include capsule endoscopy and upper endoscopy. 

Upon the current admission in late 2025, initial clinical observations recorded a blood pressure of 117/73 mmHg (reference value: <120/80 mmHg) and a respiratory rate of 18 breaths per minute (reference range: 12-20 breaths per minute). Her oxygen saturation was 95% on room air (reference range: 95-100%), while her measured temperature was 37.6°C (reference range: 36.1°C-37.2°C). Physical examination revealed a mildly distended abdomen and an uncomplicated umbilical port hernia containing fatty omental tissue. While mild abdominal tenderness was present, the patient exhibited no signs of peritonitis or rebound tenderness, and bowel sounds remained audible. 

Laboratory investigations from current admission, detailed in Table 1, revealed an elevated C-reactive protein level, accompanied by a white blood cell count that remained within the normal physiological range.

**Table 1:** Laboratory investigations upon current admission compared with physiological reference ranges

Parameter	Current admission value (late 2025)	Reference range	Interpretation
White Blood Cell (WBC) Count	9.92 x 10³/µL	4.00-11.00 x 10³/µL	Within normal limits
C-Reactive Protein (CRP)	15.73 mg/L	<5.00 mg/L	Increased
Thyroid-Stimulating Hormone (TSH)	56.97 mIU/L	0.45-4.50 mIU/L	Significantly increased
Free Thyroxine (T4)	2.23 pmol/L	9.00-19.00 pmol/L	Decreased
Serum Amylase	53.00 U/L	28-100 U/L	Within normal limits
Creatinine in Serum/Plasma	64 umol/L	45-90 umol/L	Within normal limits
Alanine Transaminase (ALT)	10 U/L	0-40	Within normal limits
Alkaline Phosphatase (ALP)	72 U/L	40-150	Within normal limits
Serum Lactate	1.76 mmol/L	0.5-2.2 mmol/L	Within normal limits
Hemoglobin	14.5 g/dL	12-16 g/dL	Within normal limits
Platelet counts	276 x 10³/µL	150-450 x 10³/µL	Within normal limits
Sodium	137 mmol/L	135-145 mmol/L	Within normal limits
Potassium	4.58 mmol/L	3.5-5 mmol/L	Within normal limits

Notably, the patient presented with severe hypothyroidism resulting from medication non-compliance, characterized by a significantly increased thyroid-stimulating hormone level and a concomitant decrease in free thyroxine. This profound endocrine dysfunction carries significant clinical relevance, as severe hypothyroidism is known to alter gastrointestinal motility and likely contributed to the patient’s reported constipation and abdominal distension.

Evaluations of renal and hepatic function, complemented by serum amylase levels, demonstrated no acute abnormalities. Laboratory investigations further supported the decision for non-operative management by demonstrating a lack of systemic toxicity or metabolic derangement. Specifically, the serum lactate level, hemoglobin and platelet counts were within normal limits (Table 1). Electrolyte panels showed no acute abnormalities. These stable biochemical parameters, when interpreted alongside the absence of progressive leukocytosis and the maintenance of hemodynamic stability, provided the clinical justification for continuing a conservative therapeutic approach.

**Figure 4:**
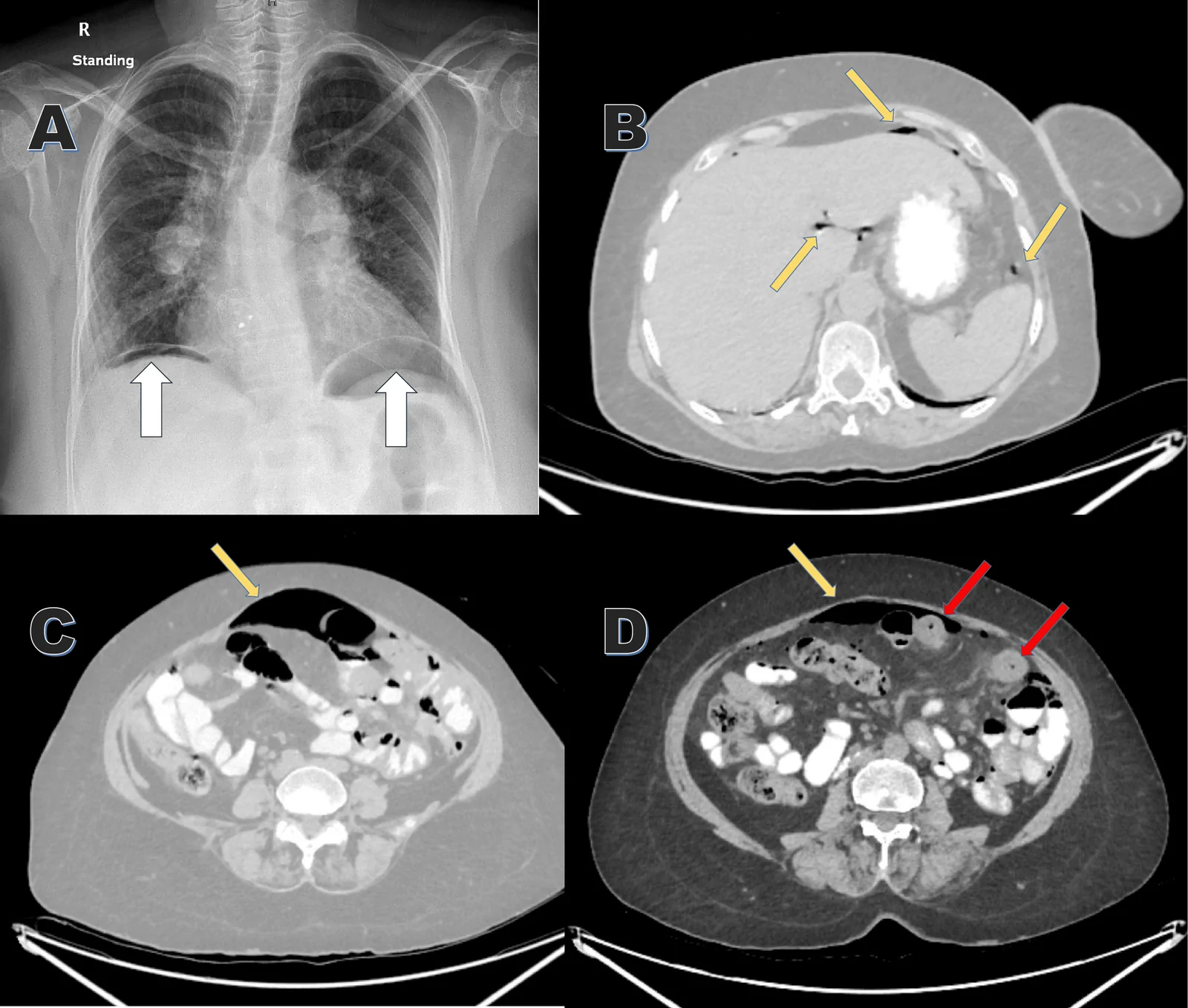
X-ray and CT images (A) X-ray study of the chest, posteroanterior (PA) view, showing air under both copulae of the diaphragm (white arrows); (B-C)  Plain computed tomography (CT) study of the abdomen axial cuts, lung window, showing evidence of pneumoperitoneum (yellow arrows); (D) Computed tomography study of the abdomen with oral contrast, axial cuts showing diffuse circumferential thickening of a long segment of the jejunum with congested mesentery (red arrows). No evidence of contrast leakage or signs of acute  enteric obstruction.

Radiological imaging studies were performed. Radiographic assessment of the chest (posteroanterior or PA view) demonstrated free gas beneath both copulae of the diaphragm. Axial non-contrast computed tomography images corroborated these findings by revealing extensive pneumoperitoneum. Following the administration of oral contrast, CT images identified diffuse circumferential wall thickening involving a long segment of the jejunum and congestion of the associated mesentery. The study yielded no evidence of contrast extravasation, which further supported the presumption that acute small intestinal obstruction was absent (Figure 4).

Given the patient’s clinical stability and the absence of contrast extravasation, a diagnosis of benign pneumoperitoneum secondary to jejunal diverticulosis was considered the most likely diagnosis. Management was strictly conservative, consisting of bowel rest (NPO), intravenous fluids, analgesics, and broad-spectrum antibiotics (piperacillin/tazobactam and metronidazole). Piperacillin with tazobactam and metronidazole were administered until an oral-contrast CT scan was performed, metronidazole was then discontinued due to no confirmation of perforation. Thyroxine replacement therapy was also reinitiated.

The patient’s clinical condition improved rapidly; she tolerated the introduction of an oral diet on the second day of hospitalization, passed stool, and remained asymptomatic during the follow-up period of her hospitalization. Serial abdominal examinations conducted by the experienced surgeon over the subsequent days of hospitalization documented a consistently soft and lax abdomen with the rapid resolution of focal tenderness. On the second day of admission, her vital signs remained stable, with a measured temperature of 37.0°C and a pulse of 82 beats per minute. No increase in inflammatory markers was observed. The intravenous antibiotic regimen was discontinued upon the patient's discharge in day four, whereas thyroxine therapy was prescribed as the sole medication for home use, due to the absence of inflammatory symptoms and the lack of an increase in inflammatory markers, as well as the patient’s significant clinical improvement.

**Figure 5:**
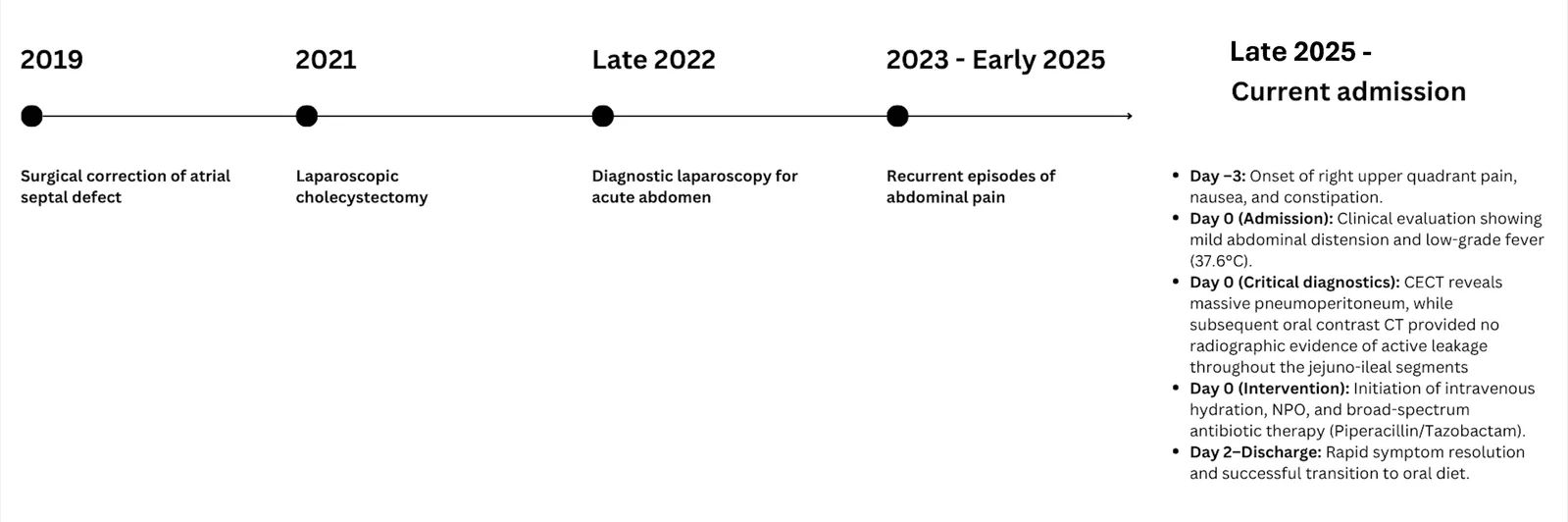
Clinical timeline of a 69-year-old patient with recurrent spontaneous pneumoperitoneum secondary to jejunal diverticulosis CECT: Contrast-Enhanced Computed Tomography; CT: Computed Tomography; NPO: łac. nil per os (bowel rest). Image credit: Created by the authors using Canva (Canva Pty Ltd., Sydney, Australia).

The comprehensive clinical timeline, illustrating the longitudinal surgical history alongside the specific sequence of the current diagnostic and therapeutic interventions, is graphically represented in Figure 5.

## Discussion

Summary and significance

The clinical presentation of pneumoperitoneum typically mandates emergent surgical examination, as it signifies a perforated hollow viscus in many cases [1,3]. However, the current case of a 69-year-old female patient highlights the diagnostic complexity of "non-surgical" or spontaneous pneumoperitoneum, a distinct entity where free intraperitoneal air exists without evidence of visceral perforation [4]. Jejunal diverticulosis, while less common than its colonic counterpart, is an increasingly recognized possible cause of recurrent SP [4]. The primary significance of this case lies in the profound diagnostic dilemma it presents to clinicians: the radiological presence of subdiaphragmatic air frequently masquerades as a surgical emergency, often leading to unnecessary and potentially morbid interventions in hemodynamically stable patients [4,6,7].

Analysis of pathophysiology

The mechanism of SP in the context of jejunal diverticulosis remains a subject of scientific speculation. These acquired "pulsion" diverticula can form through the herniation of the mucosa and submucosa at the entry points of mesenteric vessels, where the muscularis is inherently weakened [4,10]. Current theories suggest two primary pathways for gas entry into the peritoneal cavity. First, the transmural gas equilibration hypothesis proposes that the distended and thinned diverticular mucosa may act as a semipermeable membrane, allowing gas to escape without the passage of enteric contents [1,4]. Second, the occurrence of subclinical microperforations may allow for the release of air while remaining small enough to prevent significant enteric contamination or the clinical development of peritonitis [4,12]. 

Furthermore, the clinical relevance of the patient’s profound hypothyroidism, evidenced by a TSH of 56.97 mIU/L and a free T4 of 2.23 pmol/L, warrants specific consideration within the pathophysiology of her recurrent pneumoperitoneum. Thyroid hormone deficiency is established to induce significant gastrointestinal hypomotility through the accumulation of glycosaminoglycans, specifically hyaluronic acid, within the interstitial tissues and smooth muscle layers of the alimentary tract [13]. This myxomatous infiltration often results in delayed bowel transit and chronic constipation, both of which were documented in this case and likely contributed to increased intraluminal hypertension [13]. Such elevated pressures may theoretically facilitate the transmural equilibration of gas across the thinned, semipermeable mucosa of the jejunal diverticula even in the absence of a macroscopic perforation. Furthermore, clinical evidence suggests that many patients with hypothyroidism develop small intestinal bacterial overgrowth (SIBO), a condition characterized by increased gaseous volume and abdominal distension [13]. The synergy between endocrine-mediated dysmotility and the anatomical vulnerability of the jejunal diverticula provides a plausible mechanism for the patient’s recurrent episodes of SP. Identifying the impact of these comorbidities is essential, as the resolution of gastrointestinal symptoms typically depends upon the successful restoration of a euthyroid state via hormone replacement therapy [13].

Clinical context and diagnostics

Our patient’s clinical profile - characterized by hemodynamic stability, mild abdominal tenderness, and the absence of rebound tenderness - aligns with the "benign" presentation of SP reported in the literature [4]. While initial CT imaging raised suspicion of a potential duodenal perforation, the application of oral contrast CT provided additional evidence suggesting the absence of an active leak. These findings, when interpreted alongside the patient’s hemodynamic stability and the lack of peritoneal irritation, supported the decision to proceed with a conservative management strategy. This diagnostic caution is essential given that literature reports a negative laparotomy rate of 20% to 31% in patients undergoing exploration for suspected perforations [2,4,7]. Furthermore, as noted by Hoover et al., massive amounts of free air, as seen in this case, can paradoxically be a clue to a non-surgical cause, whereas surgical perforations often present with smaller, localized collections [8]. 

The decision to pursue conservative management was primarily supported by serial clinical assessment, hemodynamic stability, the absence of peritoneal signs, and the lack of radiological evidence of uncontrolled perforation. Criteria for escalation to surgical intervention included the development of overt peritonitis, hemodynamic instability, progressive leukocytosis, or radiographic evidence of perforation.

Potential non-surgical etiologies, including intrathoracic barotrauma and gynecological pathways, were excluded based on the long interval since the previous surgical procedures and the normal pelvic findings documented during the 2022 laparoscopy. The absence of metabolic derangement and the maintenance of a stable white blood cell count effectively ruled out systemic infection; moreover, the radiographic findings did not show characteristic signs of pneumatosis cystoides intestinalis.

Recommendations for tailored management

The management of jejunal diverticulosis-associated pneumoperitoneum should be a "tailored management" strategy based on the individual's clinical status rather than radiological findings alone [2,4]. Conservative management, including bowel rest, broad-spectrum antibiotics, and close monitoring, is the preferred approach for patients who are hemodynamically stable and lack overt signs of peritonitis [4]. Surgical intervention should be strictly reserved for patients demonstrating clinical deterioration, significant leukocytosis, or evidence of complications such as macro-perforation, hemorrhage, or intestinal obstruction [1,4,11]. In this patient, the recurrence of pneumoperitoneum over a three-year period further supports the safety of a non-operative approach, provided that frequent re-evaluation by senior surgical personnel is maintained [4,8].

Strengths and limitations

This case report contributes significantly to the existing body of knowledge by documenting a recurrent presentation of benign pneumoperitoneum in an elderly patient with significant comorbidities, including pulmonary hypertension and prior cardiac repair, alongside uncontrolled hypothyroidism. The findings of this study are limited by the inability to definitively confirm the exact anatomical site or pathophysiological mechanism of the gas leak and the incomplete documentation of the patient's prior clinical episodes. Furthermore, the lack of long-term follow-up resulting from patient non-compliance in conjunction with the potential confounding influence of severe hypothyroidism on gastrointestinal motility restrict the ability to draw definitive causal conclusions. Nonetheless, the case reinforces the necessity for surgeons to maintain a high index of suspicion for non-surgical causes of free air to improve patient outcomes while reducing the burden on the healthcare system [4,9].

## Conclusions

Jejunal diverticulosis represents a rare but significant consideration within the differential diagnosis of SP. While advanced imaging techniques, such as oral contrast computed tomography, may assist in the evaluation of selected stable patients, these studies cannot definitively exclude all instances of microperforation. The presence of clinical stability alongside the absence of peritoneal signs, a series of thorough physical examinations, and the absence of evidence of perforation on CT scans may support a conservative management strategy. This approach remains essential for avoiding unnecessary surgical intervention.
